# Changes in Prevalence of HIV or Syphilis among Male Sex Workers and Non-Commercial Men Who Have Sex with Men in Shenzhen, China: Results of a Second Survey

**DOI:** 10.1371/journal.pone.0167619

**Published:** 2016-12-09

**Authors:** Yuanwei Huang, Yanting Zhang, Ke Li, Jin Zhao

**Affiliations:** 1 Department of Preventive Medicine, Shantou University Medical College, Shantou, Guangdong, China; 2 Shenzhen Center for Disease Control and Prevention, Guangdong, China; National Center for AIDS/STD Control and Prevention, China CDC, CHINA

## Abstract

**Background:**

A previous time-location sampling survey (TLS) was performed in 2008 to evaluate the HIV or syphilis infection rate among male sex workers (MSWs) and non-commercial men who have sex with men (ncMSM) in Shenzhen, China. This is a second TLS performed in 2014. This article describes the findings and changes in the prevalence of HIV and syphilis.

**Methods:**

TLS was used to collect information as a second cross-sectional survey to an earlier TLS assessment. Data on behavior (e.g., sexual history and sexual behavior) were analyzed. Blood specimens were drawn for HIV and syphilis testing. To determine the changes in the prevalence of HIV and syphilis, we analyzed these results and compared them to the results of our first survey.

**Results:**

A total of 965 participants were recruited, including 489 MSWs and 476 ncMSM. Overall, the prevalence of HIV was 9.7%: 2.9% for MSWs and 16.8% for ncMSM (P<0.001). 10.9% of the 965 participants tested positive for syphilis: 4.5% among MSWs and 17.4% among ncMSM (P<0.001). The HIV prevalence in MSWs decreased from 4.5% in 2008 to 2.9% in 2014 (P = 0.143) but increased in ncMSM (7.0% in 2008 vs 16.8% in 2014, P<0.001). Decreased syphilis rates were observed in both MSWs (12.9% in 2008 vs 4.5% in 2014, P<0.001) and ncMSM (20.2% in 2008 vs 17.4% in 2014, P = 0.221).

**Conclusions:**

Overall, there was a decline in the prevalence of HIV and syphilis in MSWs but not in ncMSM. The study indicated the need for continued efforts to improve public health, particularly to counter the rising rate of HIV in ncMSM.

## Introduction

The HIV epidemic among men who have sex with men (MSM) has become a social issue in China. The prevalence of HIV infection among MSM increased from 5.98% in 2010 to 7.75% in 2014 [1, 2]. In 2015, the HIV prevalence among MSM has exceeded 8%, 27.2% of the newly diagnosed HIV-positive cases in China were attributed to MSM. Some MSM has double sexual behavior and resulted in transmitting the epidemic to general populations [3].

Shenzhen is a developed and commercialized city in southern China and is a bridge linking Hong Kong to mainland China. Because of Shenzhen’s job opportunities, the city attracts migrants, including MSM, from different parts of China. The city has more than 12 million migrants, which account for 87% of the total population [4]. The MSM population grew rapidly, with approximately 100,000 MSM living in the city [5]. The large number of MSM led to the emergence of a subgroup of MSM, male sex workers (MSWs). MSWs (also known as money boys) are hired to provide commercial sex services to males and sometimes to female clients [6]. Approximately 380,000 MSWs exist in China [7], with the number of MSWs in Shenzhen exceeding 4,000 [5]. MSWs engage repeatedly in sexual activities and are at particularly high risk of HIV infection [8]. The study conducted by Tang W. et al revealed that the prevalence of HIV and syphilis are high among MSWs in urban China [9].

Due to the high mobility of MSM population and active sexual activities in Shenzhen, to explore the changes of HIV/AIDS epidemic among MSM in this city could have great significance for China. In 2008, we first carried out a time-location sampling survey (TLS) to recruit both MSWs and non-commercial men who have sex with men (ncMSM) in Shenzhen [10]. The results indicated that MSWs and ncMSM showed differences in some sexual characteristics or behaviors [10]. This study was a second TLS conducted in 2014.This article presents an overview of current HIV prevalence and related behaviors and explores the changes in the prevalence of HIV/syphilis among MSWs and ncMSM in Shenzhen.

## Methods

### The TLS method

TLS is a venue-based sampling based on clusters. In TLS, a primary-sampling unit is defined as the combination of site and time, and the same site may be included in the sampling frame more than once, at different times of the day or week. Detailed methods were described previously [4, 5]. The method has been used to sample different high-risk groups [4, 5, 11].

### Data collection

The parallel recruitment was conducted from June to November 2014. All the venues (e.g. bars, massage centers and saunas) providing sex for MSM were surveyed. In total, 46 MSM venues were included in our sampling frame, with 20 selected per month. As described in detailed previously [10], we recruited MSWs with the following criteria: (1) > 18 years old, (2) lived in Shenzhen for more than 1 month at the time of survey and (3) provided commercial sex services to males in the previous month. The inclusion criteria for MSM were (1) having homosexual activities in Shenzhen in the previous 6 months and not having been paid for male–male sex.

Eligible participants had to sign a consent form at the start of survey, then they were invited to complete a platform-assisted questionnaire. The questionnaire collected data on demographics and sexual behaviors (S1 and S2 Tables). After the questionnaire interview, blood samples were taken by staff of the Shenzhen Center for Disease Control and Prevention (CDC) for HIV and syphilis testing.

As described in detailed previously [5, 10]. The testing was performed according to standard procedures provided by the Shenzhen CDC laboratory. HIV was tested by using a rapid test (Determine HIV-1/2/O, Abbott Laboratories, IL) and enzyme linked immunosorbent assay (Wantai Biotech Inc., Beijing) for screening and western blot analysis (Genlabs Diagnostics, Singapore) for confirmation. Syphilis was tested with the rapid plasma regain method (Rongsheng Biotech Inc., Shanghai) for qualitative screening and Treponema pallidum particle agglutination assay (Fujirebio Inc, Japan) for confirmation.

### Data Analysis

Descriptive analysis was undertaken using SPSS 17.0 (SPSS Inc., Chicago, IL) to compare social and sexual behavioral characteristics of MSWs and ncMSM. Pearson chi-square, or nonparametric tests as applicable, were performed to find out factors related to HIV infection in the 2 groups. Then, we used multiple logistic regression analysis to determine the association between risk factors and HIV-syphilis infection in MSWs and MSM. Variables included in the analysis were from prior knowledge and from the results of univariate analysis (P<0.2). For the MSWs, we added 3 additional variables: number of commercial male clients, selling sex to women and duration of sex work. P<0.05 was considered statistically significant (S3 Table).

### Ethics approve and informed consent

The study was approved by the Internal Review Board of the Shenzhen Center for Disease Control and Prevention (2012006). Written informed consent was obtained from all participants included in the study.

## Results

### HIV/Syphilis/sexually transmitted infections

We recruited 965 eligible participants (489 MSWs and 476 ncMSM). A total of 94 were HIV-seropositive (9.7%; 95% confidence interval [CI] 7.9–11.6%]): 2.9% for MSWs (95%CI: 1.4–4.3%) and 16.8% for ncMSM (95%CI 13.5–20.2%) (P<0.001). Overall, 105 participants were syphilis-positive (10.9%; 95%CI 8.9–12.9%): 4.5% for MSWs (95%CI 2.7–6.3%) and 17.4% for ncMSM (95%CI 14.0–20.9%) (P<0.001). A large proportion of both MSWs and ncMSM who were HIV-positive were also syphilis-positive (18.2% for MSWs and 42.2% for ncMSM). There was a significant greater proportion of ncMSM reporting ever having being diagnosed with sexually transmitted infections (STIs) (9.7% for ncMSM and 0.8% for MSWs) (**Table 1**).

**Table 1:** HIV infection and demographic/sexual behavior characteristics in MSWs and ncMSM.

	Participants, n(%)		P	HIV-positive, n(%^2^)	
	MSWs(n = 489)	ncMSM(n = 476)		MSWs(n = 14)	ncMSM(n = 80)
HIV-positive	14(2.9)	80(16.8)	<0.001^1^		
Syphilis-positive			<0.001^1^		
Yes	22(4.5)	83(17.4)		4(18.2)^3^	35(42.2)^3^
No	467(95.5)	393(82.6)		10(2.1)	45(11.5)
Ever diagnosed with STIs			<0.001^1^		
Yes	4(0.8)	46(9.7)		1(25.0)^4^	8(17.4)
No	485(99.2)	430(90.3)		13(2.7)	72(16.7)
Age, years			<0.001^5^		
<20	17(3.5)	6(1.2)		1(5.9)	1(16.7)^6^
20–29	443(90.6)	196(41.2)		12(2.7)	25(12.8)
≥30	29(5.9)	274(57.6)		1(3.4)	54(19.7)
Length of stay in Shenzhen, years			<0.001^5^		
<1	119(24.3)	111(23.3)		4(3.4)	22(19.8)
1–2	237(48.5)	74(15.6)		6(2.5)	17(23.0)
>2	133(27.2)	291(61.1)		4(3.0)	41(14.1)
Venue type of recruitment			<0.001^1^		
Bar	315(64.4)	112(23.5)		7(2.2)^4^	7(6.3)^3^
Dorm-based venue	161(32.9)	102(21.4)		5(3.1)	10(9.8)
Recreational center	10(2.1)	175(36.8)		1(10.0)	46(26.3)
Sauna	3(0.6)	87(18.3)		1(33.3)	17(19.5)
Education			<0.001^5^		
Junior high school or less	117(23.9)	102(21.4)		6(5.1)^7^	26(25.5)^6^
Senior high school	287(58.7)	161(33.8)		7(2.4)	34(21.1)
College or above	85(17.4)	213(44.7)		1(1.2)	20(9.4)
Employment			<0.001^1^		
Unemployed/part-time/retired	37(7.6)	67(14.1)		1(2.7)	11(16.4)
Full-time employed	451(92.2)	397(83.4)		13(2.9)	67(16.9)
Student	1(0.2)	12(2,5)		0(0)	2(16.7)
Monthly income, RMB			<0.001^5^		
≤3000	66(13.5	108(22.7)		4(6.1)	20(18.5)^6^
3001–5000	168(34.4)	234(49.2)		5(3.0)	44(18.8)
>5000	255(52.1)	134(28.1)		5(2.0)	16(11.9)
Marital status			<0.001^1^		
Married	20(4.1)	135(28.4)		2(10.0)	28(20.7)
Unmarried	469(95.9)	341(71.6)		12(2.4)	52(15.2)
Sexual orientation			<0.001^1^		
Gay	312(63.8)	319(67.0)		10(3.2)	44(13.8)^4^
Bisexual	42(8.6)	105(22.1)		0(0)	26(24.8)
Heterosexual or unsure	135(27.6)	52(10.9)		4(3.0)	10(19.2)
Anal sex role			<0.001^1^		
Insertive only	218(44.6)	188(39.5)		7(3.2)^4^	25(13.3)
Receptive only	23(4.7)	67(14.1)		3(13.0)	10(14.9)
Both	248(50.7)	221(46.4)		4(1.6)	45(20.4)
Gender of the first sexual partner			0.141^1^		
Male	355(72.6)	325(68.3)		10(2.8)	43(13.2)^4^
Female	134(27.4)	151(31.7)		4(3.0)	3(24.5)
Age at sex debut, years			0.009^6^		
≤18	127(26.0)	69(14.5)		2(1.6)	11(15.9)
19–25	349(71.4)	305(64.1)		10(2.9)	48(15.7)
≥26	13(2.7)	102(21.4)		2(15.4)	21(20.6)
Male sex partner^8^			0.010^5^		
0	158(32.3)	71(14.9)		4(2.5)	13(18.3)
1	62(12.7)	138(29.0)		2(3.2)	16 (11.6)
>1	269(55.0)	267(56.1)		8(3.0)	51(19.1)
Female sex partner^8^			<0.001^5^		
0	426(87.1)	436(92.2)		12(2.8)	73(16.7)
1	19(3.9)	34(7.2)		1(5.3)	7(20.6)
>1	44(9.0)	3(0.6)		1(2.3)	0(0)
Had one-night stand with male partner^8^			0.256^1^		
Yes	296(60.5)	305(64.1)		7(2.4)	58(19.0)
No	193(39.5)	171(35.9)		7(3.6)	22(12.9)
Group sex^8^			0.020^1^		
Yes	36(7.4)	56(11.8)		0(0)	13(23.2)
No	453(92.6)	420(88.2)		14(3.1)	67(16.0)
Commercial male partner^8^			-		
≤4	21(43.1)	-		8(3.8)	-
>4	278(56.9)	-		6(2.2)	-
Sold sex to women^8^			-		
Yes	37(7.6)	-		0(0)	-
No	452(92.4)	-		14(3.1)	-
Duration of sex work, years			-		
<1	135(27.6)	-		1(0.7)	-
1–3	313(64.0)	-		10(3.2)	-
>3	41(8.4)	-		3(7.3)	-
Condom use with male sex partner^8^			0.001^1^		
Yes	400(81.8)	348(73.1)		8(2.0)^4^	61(17.5)
No	89(18.2)	128(26.9)		6(6.7)	19(14.8)
Condom use with female sex partner^8^			<0.001^1^		
Yes	53(10.8)	20(4.2)		2(3.8)	5(25.0)
No	436(89.2)	456(95.8)		12(2.8)	75(16.4)
Ever used illicit drugs			<0.001^1^		
Yes	128(26.2)	189(39.7)		6(4.7)	24(12.7)
No	361(73.8)	287(60.3)		8(2.2)	56(19.5)
Had a previous HIV test			<0.001^1^		
Yes	373(76.3)	306(64.3)		7(1.9)^4^	36(11.8)^3^
No	116(23.7)	170(35.7)		7(6.0)	44(25.9)

^1^ Two-sided chi-square test of proportions

^2^ HIV-positive rate

^3^ P<0.001 and

^4^ P<0.05 by chi-square test of rate of HIV infection in different categories of MSWs and ncMSM

^5^ Non-parametric test

^6^ P<0.001 and

^7^ P<0.05 by non-parametric test of rate of HIV infection in different categories of MSWs and ncMSM

^8^ Within 6 months

STI, sexually transmitted infection

### Sociodemographic Characteristics

All sociodemographic characteristics differed between MSWs and ncMSM (**Table 1**). NcMSM had an older age (≥30 years) (57.6% for ncMSM and 5.9% for MSWs) and generally had lived in Shenzhen for a longer time (>2 years) (61.1% for ncMSM and 27.2% for MSWs) compared to MSWs. MSWs tended to have higher monthly income. A large proportion of both MSWs and ncMSM who were HIV-positive had a low level of education (5.1% for MSWs and 25.5% for ncMSM) as did a large proportion of ncMSM with older age (>30 years old) (19.7% for ncMSM and 6.9% for MSWs), low monthly income (≤3000,RMB) [18.5% for ncMSM and 6.1% for MSWs] and self-identified as bisexual (24.8% for ncMSM and 0% for MSWs).

### Sexual Practices

Both similarities and differences in sexual behaviors were observed in the 2 compared groups (**Table 1**). 63.8% MSWs and 67.0% ncMSM self-identified as gay or homosexual. In the previous 6 months, 55.0% MSWs and 56.1% ncMSM had more than one male sex partner. A greater propotion of both MSWs (60.5%) and ncMSM (64.1%) had one-night-stand sex with a male partner. 7.4% of MSWs and 11.8% of ncMSM reported engaging in group sex. A slightly higher proportion of MSWs than ncMSM reported condom use during male sex (81.8% vs 73.1%), and more MSWs than ncMSM reported the use of condoms during female sex (10.8% for MSWs and 4.2% for ncMSM). More ncMSM than MSWs reported having used illicit drugs (39.7% vs 26.2%). In addition, 64.0% of MSWs engaged in sex work for 1 to 3 years, 8.4% longer than 3 years. 56.9% MSWs reported having more than 4 male clients, but only a few (7.6%) had sold sex to women in the past 6 months.

For MSWs, HIV infection rates were high for those with receptive anal intercourse or not using condoms during male sex and previous HIV test. HIV infection rates were high for ncMSM who reported their first sex partner as female and no previous HIV test.

### The comparisons with previous survey

Table 2 presents the HIV and syphilis infection rate for each group and compares with this data with the previous data collected in 2008. The syphilis-positive rate in MSWs significantly decreased [12.9% in 2008 vs 4.5% in 2014 (P<0.001)]. In addition, the HIV-infection rate in MSWs [4.5% in 2008 vs 2.9% in 2014 (P = 0.143)] and the syphilis-positive rate in ncMSM [20.2% in 2008 vs 17.4% in 2014 (P = 0.221)] also decreased. However, a significantly increased HIV-infection rate was observed in ncMSM [7.0% in 2008 vs 16.8% in 2014 (P<0.001)].

**Table 2:** A comparison with the data from the previous survey.

	MSWs, n(%)		P	ncMSM, n(%)		P
	Survey in 2008(n = 850)	Survey in 2014(n = 489)		Survey in 2008(n = 801)	Survey in 2014(n = 476)	
HIV-positive			0.143^1^			0.000^1^
Yes	38(4.5)	14(2.9)		56(7.0)	80(16.8)	
No	812(95.5)	475(97.1)		745(93.0)	396(83.2)	
Syphilis-positive			0.000^1^			0.221^1^
Yes	110(12.9)	22(4.5)		162(20.2)	83(17.4)	
No	740(87.1)	467(95.5)		639(79.8)	393(82.6)	

TLS, time-location sampling survey

^1^ Two-sided chi-square test of proportions

### Factors correlated with HIV infection of MSWs and ncMSM

In the univariate analyses (unadjusted models) (Table 3), HIV infection among MSWs was positively associated with syphilis infection (OR = 10.2, 95%CI: 2.9–35.5), previous HIV testing (OR = 3.4; 95%CI: 1.2–9.8), taking both receptive and insertive anal sex role (OR = 9.2; 95%CI: 1.9–43.8),and reporting ever having being diagnosed with STIs (OR = 12.1; 95%CI: 1.2–124.3). but condom use in male–male sex was less likely to be HIV infection (OR = 0.3; 95%CI: 0.1–0.8). For ncMSM, syphilis infection (OR = 5.6; 95%CI: 3.3–9.6), receiving a higher education (college above) (OR = 2.6; 95%CI: 1.4–4.7) and first sex with male partner (OR = 2.1; 95%CI: 1.3–3.4) were associated with HIV-positivity, but previous HIV testing (OR = 0.4; 95%CI: 0.2–0.6) and elder in age (>30) (OR = 0.6; 95%CI: 0.4–0.9) were negatively associated with HIV infection.

**Table 3:** Estimated HIV risk/prevention behavior in MSWs and ncMSM.

	MSWs(n = 489)				ncMSM(n = 476)			
	COR(95%CI)	P	AOR(95%CI)	P	COR(95%CI)	P	AOR(95%CI)	P
Syphilis infection	**10.2(2.9,35.5)**	**0.000**	**9.0(2.4,33.5)**	**0.001**	**5.6(3.3,9.6)**	**0.000**	**5.9(3.2,10.9)**	**0.000**
Ever diagnosed with STIs	**12.1(1.2,124,3)**	**0.036**	**13.9(1.5,134.6)**	**0.045**	1.0(0.5,2.3)	0.911	1.3(0.5,3.4)	0.557
Tested for HIV	**3.4(1.2–9.8)**	**0.026**	2.8(0.7,11.1)	0.152	**0.4(0.2,0.6)**	**0.000**	**0.4(0.2,0.7)**	**0.001**
Condom use with male sex partner	**0.3(0.1,0.8)**	**0.022**	**0.3(0.1,0.9)**	**0.046**	0.8(0.5,1.4)	0.488	0.7(0.4,1.5)	0.485
Anal sex role(both)	**9.2(1.9,43.8)**	**0.006**	3.6(0.4,32.3)	0.251	0.7(0.3,1.4)	0.323	-	-
Elder in age(>30)	0.3(0.1,1.6)	0.179	0.3(0.1,2.6)	0.280	**0.6(0.4,0.9)**	**0.048**	0.6(0.3,1.2)	0.181
Higher education(college above)	2.1(0.3,0.3,17.3)	0.491	7.6(0.4,160.9)	0.191	**2.6(1.4,4.7)**	**0.002**	**2.1(1.0,4.2)**	**0.049**
First sex partner(male)	1.1(0.3,3.4)	0.921	5.2(0.9,31.7)	0.072	**2.1(1.3,3.4)**	**0.003**	**2.1(1.2,3.9)**	**0.014**

OR, odds ratio

COR, crude OR

Variables included in the multiple logistic regression model: HIV/syphilis infection, age, education, sex role, history of HIV test, history of STIs, condom use, first sex partner

AOR, adjusted OR, adjusted for other variables listed in the text

STI, sexually transmitted infection

Significant results are in bold (P<0.05)

In multivariate regression analysis (**Table 3**), most of the HIV associations remained significant. Syphilis infection (adjusted OR [aOR] = 9.0; 95%CI: 2.4–33.5) and reporting ever having being diagnosed with STIs (aOR = 13.9; 95%CI: 1.5–134.6) were independently associated with HIV infection in MSWs, but condom use in male–male sex (aOR = 0.3; 95%CI: 0.1–0.9) was protective. Syphilis infection (aOR = 5.9; 95%CI: 3.2–10.9), having a higher education (college above) (aOR = 2.1; 95%CI: 1.0–4.2) and first sex with male partner (aOR = 2.1; 95%CI: 1.2–3.9) were positively associated with HIV infection in ncMSM, but having had an HIV test (aOR = 0.4; 95%CI: 0.2–0.7) was a protective factor.

In logistic regression analyses evaluating the risk factors for combined HIV infection (**Table 4**), syphilis infection, reporting ever having being diagnosed with STIs, having a higher monthly income and being married were associated with HIV-positivity. After controlling for MSM type, syphilis infection and having a higher education such as senior high school, college or above were associated with an increased HIV infection.

**Table 4:** Factors correlated with combined HIV infection.

Characteristics	HIV+/total	Univariate		Adjusted for MSM type	
		OR(95%CI)	P	OR(95%CI)	P
Syphilis-positive					
No	55/860	1		1	
Yes	39/105	8.6(5.3,14.0)	0.000	6.1(3.7,10.1)	0.000
Ever diagnosed with STIs					
No	85/915	1		NS	
Yes	9/50	2.1(1.0,4.6)	0.048	NS	
Age, years					
<20	2/23	1		1	
20–29	36/639	0.4(0.1,1.8)	0.250	NS	
≥30	56/303	0.3(0.2,0.4)	0.000	0.6(0.3,0.9)	0.024
Education					
Junior high school or less	32/219	1		1	
Senior high school	41/448	2.3(1.3,4.0)	0.006	3.5(1.9,6.4)	0.000
College or above	21/298	1.3(0.8,2.3)	0.309	2.4(1.4,4.4)	0.002
Employment					
Full-time employed	80/848	1		1	
Unemployed/part-time/retired	12/104	0.6(0.1,2.6)	0.474	NS	
Student	2/13	0.7(0.1,3.6)	0.688	NS	
Monthly income, RMB					
≤3000	24/174	1		1	
3001–5000	49/402	2.8(1.5,5.2)	0.001	NS	
>5000	21/389	2.4(1.4,4.1)	0.001	NS	
Marital status					
Unmarried	64/810	1		1	
Married	30/155	2.8(1.7,4.5)	0.000	NS	
Sexual orientation					
Gay	54/631	1		1	
Biosexual	26/147	1.2(0.6,2.1)	0.641	NS	
Heterosexual or unsure	14/187	2.7(1.3,5.3)	0.006	NS	
Anal sex role					
Insertive only	32/406	1		1	
Receptive only	13/90	0.7(0.5,1.2)	0.193	NS	
Both	49/469	1.4(0.7,2.8)	0.217	NS	
Gender of first sexual partner					
Female	41/285	1		1	
Male	53/680	0.5(0.3,0.7)	0.002	0.5(0.3,0.8)	0.005
Age at sex debut, years					
≤18	15/196	1		1	
19–25	58/653	0.4(0.2,0.8)	0.006	NS	
≥26	21/115	0.4(0.3,0.8)	0.003	NS	
Male sex partner^1^					
0	17/229	1		1	
1	18/200	0.6(0.4,1.1)	0.132	NS	
>1	59/536	0.8(0.5,1.4)	0.429	NS	
Female sex partner^1^					
0	85/865	1		1	
1	8/53	5.0(0.7,36.8)	0.113	NS	
>1	1/47	8.2(0.9,68.1)	0.052	NS	
Had one-night stand with male partner^1^					
No	29/364	1		1	
Yes	65/601	1.4(0.9,2.2)	0.148	NS	
Group sex^1^					
No	81/873	1		1	
Yes	13/92	1.6(0.9,3.0)	0.135	NS	
Condom use with male sex partner^1^					
No	25/217	1		1	
Yes	69/748	0.8(0.5,1.3)	0.315	NS	
Condom use with female sex partner^1^					
No	87/892	1		1	
Yes	7/73	0.9(0.4,2.2)	0.964	NS	
Ever used illicit drugs					
No	64/648	1		1	
Yes	30/317	1.0(0.6,1.5)	0.839	NS	
Had a previous HIV test					
No	51/543	1		1	
Yes	43/422	1.1(0.7,1.7)	0.679	0.5(0.3,0.8)	0.006

^1^ Within 6 months

OR, odds ratio

NS, not significant

## Discussion

The current study provides updated data about HIV and syphilis prevalence for MSWs and ncMSM. Our finding confirmed that the prevalence of HIV and syphilis were reduced in MSWs but not in ncMSM.

For MSWs, the overall prevalence of HIV decreased from 4.5% to 2.9% and the syphilis-positive rate decreased from 12.9% to 4.5% compared with 2008. The prevalence of HIV and syphilis in MSWs in Shenzhen were lower than MSWs in other parts in China [12]. Many factors may contribute to the reduction in the prevalence of HIV among MSWs but not among ncMSM. First, condom use in MSWs was increased. In the survey, we found a higher rate of condom use by MSWs than ncMSM during male sex. Therefore, the risk of HIV infection was reduced by this protective behavior. Second, because the sole aim of sex for most MSWs was to earn money rather than pursuit of sexual gratification, MSWs were more likely to adopt protective behavior compared with ncMSM, thus protecting their health and decreasing their risk of infection.

For MSWs, HIV-positivity was positively associated with previous HIV testing by logistic regression analysis. The reasons for higher HIV infection rate in those HIV testers may be explained as follows. MSWs engaged in high-risk work. Due to self-protection awareness and were more likely to be knowledgeable about AIDS [13], these workers were more willing to undergo frequent HIV testing. We found more MSWs underwent HIV testing compared with ncMSM. However, the study showed that repeat testers are more likely to engage in high-risk sexual behavior than non-repeat testers [13]. So for MSWs, frequent testing might be associated with risky behaviors (sex work). In contrast, we found a higher HIV infection rate in ncMSM who reported no previous HIV testing. This is a significant problem, as non-testers may not be aware of their HIV infection status, and infected non-testers can unknowingly transmit the disease to their partners [14], thereby leading to a high rate of HIV infection.

For ncMSM, a notable finding was that the HIV-infection rate increased from 7.0% to 16.8%. The HIV prevalence in ncMSM in Shenzhen was higher than ncMSM in other regions in China [9, 15]. Overall, HIV has continued to spread rapidly among MSM in China [16, 17]. Many ncMSM in our study have been infected elsewhere but were tested and found to be positive in Shenzhen. For one thing, most MSM are not Shenzhen natives (in our study, 19.8% infected ncMSM living in Shenzhen for less 1 year, 23.0% of these people living in this city for 1 to 2 years), they come from different parts of China. NcMSM usually have male-male sex elsewhere and then possibly continue homosexual activities in Shenzhen. In contrast to ncMSM, MSWs may provide commercial sex services only in Shenzhen or start their sex work career in Shenzhen. Once MSWs have earned enough money, they may stop this work and/or leave Shenzhen. Therefore, the HIV-infection risk in ncMSM was much higher than in MSWs. Our study found that ncMSM were more likely than MSWs to be HIV- or syphilis-positive. Additionally, most ncMSM do not stay in Shenzhen for a long time. Due to high liquidity of the population and the difficulty in identification of ncMSM (due to social stigma), there are few effective intervention strategies to target this population. For this reason, the spread of HIV in ncMSM may not be well-controlled.

We found that the syphilis positive rate in ncMSM decreased from 20.2% to 17.4% compared with the results of the previous survey in 2008. The prevalence of syphilis was 10.9% among MSM (95% CI: 9.8–12.1%) during 2010 to 2013 in China [18]. Therefore, the syphilis infection rate in Shenzhen maintains at a stable level, but shows a general tendency of decline. In addition, the results indicated a higher rate of HIV infection for ncMSM who were syphilis-positive. Because of the shared transmission routes of the 2 infections and because of adverse interactions–syphilis facilitates both HIV transmission and acquisition [19]–the co-infection rate of syphilis and HIV is high. HIV can alter the clinical manifestations of syphilis and, in turn, syphilis induces immune activation and favors viral replication, so it may accelerate HIV transmission [20, 21]. Syphilis–HIV co-infection remains a major public health issue, especially among MSM [22].

There are limitations of this study. Our study was not a cohort study, but two cross-sectional studies. The included population may vary between the two studies, precluding direct comparison of HIV infection rate and behavioral changes. However, due to the high liquidity and instability of the MSWs and ncMSM populations in Shenzhen, performing a cohort study was impossible. Therefore, a series of cross-sectional studies was a reasonable alternative.

In summary, compared with the data from the previous survey in 2008, both the HIV-infection and syphilis-positive rates were decreased in MSWs. Additionally, the syphilis-positive rate in ncMSM was also decreased. However, we detected a possible increasing prevalence of HIV among ncMSM. The rising rate of HIV in ncMSM show targeted interventions for this group still need to be strengthened and improved to combat this public health problem.

## Supporting Information

S1 Table The survey questions in Chinese. (DOCX)

S2 Table The survey questions in English. (DOCX)

S3 Table The dataset for analysis. (XLS)
