# Correction: Changes in Prevalence of HIV or Syphilis among Male Sex Workers and Non-Commercial Men Who Have Sex with Men in Shenzhen, China: Results of a Second Survey

**DOI:** 10.1371/journal.pone.0175017

**Published:** 2017-03-28

**Authors:** Yuanwei Huang, Yanting Zhang, Ke Li, Jin Zhao

There are errors in Tables 3 and 4, and corresponding errors in the Results section under the subheading “Factors correlated with HIV infection of MSWs and ncMSM.” Please see the corrected Tables 3 and 4 here.

**Table 3:** Estimated HIV risk/prevention behavior in MSWs and ncMSM.

	MSWs (n = 489)				ncMSM (n = 476)			
	COR (95%CI)	*P*	AOR (95%CI)	*P*	COR (95%CI)	*P*	AOR (95%CI)	*P*
Syphilis infection	**10.2 (2.9,35.5)**	**0.000**	**9.0 (2.4,33.5)**	**0.001**	**5.6 (3.3,9.6)**	**0.000**	**5.9 (3.2,10.9)**	**0.000**
Ever diagnosed with STIs	**12.1 (1.2,124,3)**	**0.036**	**13.9 (1.5,134.6)**	**0.045**	1.0 (0.5,2.3)	0.911	1.3 (0.5,3.4)	0.557
Tested for HIV	**3.4 (1.2–9.8)**	**0.026**	2.8 (0.7,11.1)	0.152	**0.4 (0.2,0.6)**	**0.000**	**0.4 (0.2,0.7)**	**0.001**
Condom use with male sex partner	**0.3 (0.1,0.8)**	**0.022**	**0.3 (0.1,0.9)**	**0.046**	0.8 (0.5,1.4)	0.488	0.7 (0.4,1.5)	0.485
Anal sex role (both)	**9.2 (1.9,43.8)**	**0.006**	3.6 (0.4,32.3)	0.251	0.7 (0.3,1.4)	0.323	-	-
Younger in age(<20)	0.3 (0.1,1.6)	0.179	0.3 (0.1,2.6)	0.280	**0.6 (0.4,0.9)**	**0.048**	0.6 (0.3,1.2)	0.181
Lower education (Junior high school or less)	2.1 (0.3,17.3)	0.491	7.6 (0.4,160.9)	0.191	**2.6 (1.4,4.7)**	**0.002**	**2.1 (1.0,4.2)**	**0.049**
First sex partner (male)	1.1 (0.3,3.4)	0.921	5.2 (0.9,31.7)	0.072	**2.1 (1.3,3.4)**	**0.003**	**2.1 (1.2,3.9)**	**0.014**

OR, odds ratio

COR, crude OR

Variables included in the multiple logistic regression model: HIV/syphilis infection, age, education, sex role, history of HIV test, history of STIs, condom use, first sex partner

AOR, adjusted OR, adjusted for other variables listed in the text

STI, sexually transmitted infection

Significant results are in bold (P<0.05)

**Table 4:** Factors correlated with combined HIV infection.

Characteristics	HIV+/total	Univariate		Adjusted for MSM type	
		OR (95%CI)	*P*	OR (95%CI)	*P*
Syphilis-positive					
No	55/860	1		1	
Yes	39/105	8.6 (5.3,14.0)	0.000	6.1 (3.7,10.1)	0.000
Ever diagnosed with STIs					
No	85/915	1		NS	
Yes	9/50	2.1 (1.0,4.6)	0.048	NS	
Age, years					
<20	2/23	1		1	
20–29	36/639	0.6 (0.1,2.7)	0.539	NS	
≥30	56/303	2.3 (0.5,10.4)	0.250	NS	
Education					
Junior high school or less	32/219	1		1	
Senior high school	41/448	0.6 (0.4,0.9)	0.035	NS	
College or above	21/298	0.4 (0.2,0.8)	0.006	0.3 (0.2,0.5)	0.000
Employment					
Full-time employed	80/848	1		1	
Unemployed/part-time/retired	12/104	1.3 (0.7,2.4)	0.494	NS	
Student	2/13	1.7 (0.4,8.0)	0.474	NS	
Monthly income, RMB					
≤3000	24/174	1		1	
3001–5000	49/402	0.9 (0.5,1.5)	0.595	NS	
>5000	21/389	0.4 (0.2,0.7)	0.001	NS	
Marital status					
Unmarried	64/810	1		1	
Married	30/155	2.8 (1.7,4.5)	0.000	NS	
Sexual orientation					
Gay	54/631	1		1	
Biosexual	26/147	2.3 (1.4,3.8)	0.001	1.8 (1.1,3.0)	0.033
Heterosexual or unsure	14/187	0.9 (0.5,1.6)	0.641	NS	
Anal sex role					
Insertive only	32/406	1		1	
Receptive only	13/90	1.9 (0.9,3.9)	0.053	NS	
Both	49/469	1.4 (0.9,2.2)	0.217	NS	
Gender of first sexual partner					
Female	41/285	1		1	
Male	53/680	0.5 (0.3,0.7)	0.002	0.5 (0.3,0.8)	0.005
Age at sex debut, years					
≤18	15/196	1		1	
19–25	58/653	1.2 (0.6,2.1)	0.591	NS	
≥26	21/115	2.7 (1.3,5.4)	0.006	NS	
Male sex partner^1^					
0	17/229	1		1	
1	18/200	1.2 (0.6,2.5)	0.552	NS	
>1	59/536	1.5 (0.9,2.7)	0.132	NS	
Female sex partner^1^					
0	85/865	1		1	
1	8/53	1.6 (0.7,3.5)	0.222	NS	
>1	1/47	0.2 (0.1,1.5)	0.113	NS	
Had one-night stand with male partner^1^					
No	29/364	1		1	
Yes	65/601	1.4 (0.9,2.2)	0.148	NS	
Group sex^1^					
No	81/873	1		1	
Yes	13/92	1.6 (0.9,3.0)	0.135	NS	
Condom use with male sex partner^1^					
No	25/217	1		1	
Yes	69/748	0.8 (0.5,1.3)	0.315	NS	
Condom use with female sex partner^1^					
No	87/892	1		1	
Yes	7/73	0.9 (0.4,2.2)	0.964	NS	
Ever used illicit drugs					
No	64/648	1		1	
Yes	30/317	1.0 (0.6,1.5)	0.839	NS	
Had a previous HIV test					
No	51/543	1		1	
Yes	43/422	1.1 (0.7,1.7)	0.679	0.5 (0.3,0.8)	0.006

^1^Within 6 months

OR, odds ratio

NS, not significant

The last sentence of the first paragraph of the Results section under the subheading “Factors correlated with HIV infection of MSWs and ncMSM” should be: For ncMSM, syphilis infection (OR = 5.6; 95%CI: 3.3–9.6), receiving a lower education (Junior high school or less) (OR = 2.6; 95%CI: 1.4–4.7) and first sex with male partner (OR = 2.1; 95%CI: 1.3–3.4) were associated with HIV-positivity. Previous HIV testing (OR = 0.4; 95%CI: 0.2–0.6) and younger in age (<20) (OR = 0.6; 95%CI: 0.4–0.9) were negatively associated with HIV infection.

The last sentence of the second paragraph of the Results section under the subheading “Factors correlated with HIV infection of MSWs and ncMSM” should be: Syphilis infection (aOR = 5.9; 95%CI: 3.2–10.9), having a lower education (Junior high school or less) (aOR = 2.1; 95%CI: 1.0–4.2) and first sex with male partner (aOR = 2.1; 95% CI: 1.2–3.9) were positively associated with HIV infection in ncMSM, but having had an HIV test (aOR = 0.4; 95%CI: 0.2–0.7) was a protective factor.

The first sentence of the third paragraph of the Results section under the subheading “Factors correlated with HIV infection of MSWs and ncMSM” should be: In logistic regression analyses evaluating the risk factors for combined HIV infection (Table 4), syphilis infection, reporting ever having being diagnosed with STIs, people who are bisexual, age at sex debut (≥26) and being married were associated with HIV-positivity. After controlling for MSM type, syphilis infection and people who are bisexual were associated with an increased HIV infection.
