# Supplementary material for: Changes in Prevalence of HIV or Syphilis among Male Sex Workers and Non-Commercial Men Who Have Sex with Men in Shenzhen, China: Results of a Second Survey
Source: PLoS One. 2016 Dec 9;11(12):e0167619. doi: 10.1371/journal.pone.0167619 (PMC5147921; doi:10.1371/journal.pone.0167619)
Supplement: S1 Table — (DOCX) [file pone.0167619.s001.docx]

S1 Table The survey questions in chinese

| A01问卷编号：＿＿＿­＿ |
| --- |
| A02 调查日期：＿＿＿­＿ |
| A03 样本来源：①酒吧②歌舞厅③茶室④会所⑤浴池⑥桑拿⑦足疗⑧按摩⑨其他（请注明＿＿＿­＿） |
| 在调查前，我们想确定你是否适合做这个调查，在过去六个月，你曾否在深圳或者香港有过男男同性性行为(包括肛交或口交)?①没有（非合适受访者）②有（继续访问） |
| B01 出生日期：＿＿＿­＿年＿＿月＿＿日 |
| B02婚姻状况：①未婚②已婚 |
| B03 户籍所在地：      ①深圳 ②本省③外省（请注明＿＿＿­＿）④外籍（请注明＿＿＿­＿） |
| B04 民族：＿＿＿­＿ |
| B05 在深圳居住时间： ①<3个月 ②3-6月③7-12月④1-2年 ⑤2年以上 |
| B06 文化程度： ①文盲②小学③初中④高中或中专⑤大专及以上 |
| B07您目前工作状况：①全职 ②兼职③待业/失业 ④退休⑤自雇/自营⑥学生⑦其它(请注明) |
| B08目前您的月收入水平：①无收入 ②1000以下③1001－2000 ④2001－3000 ⑤3001－4000 ⑥4001－5000⑦5001－7000⑧7000以上 |
| B09您目前是否居住在深圳？ ①是②否 |
| C01.您如何评价自己现阶段的性取向？①同性恋②异性恋③双性恋④不确定 |
| C02.您第一次发生性行为（包括与男性或女性的口交，阴道交，肛交）的年龄是？＿＿周岁 |
| C03.您第一个性伴是男性还是女性？①男②女 |
| C04最近6个月，你曾处过多少个确立关系的男朋友（BF）？______个（没有请填 0） |
| C05最近6个月你与男朋友之间的性生活频率如何：平均每月________次 |
| C06最近6个月，您有过多少个一夜情的男性性伴？________个（没有请填 0） |
| C07在最近6个月内，你参与过多少次群交性行为（三人或三人以上）？________次（没有请填 0） |
| C08最近六个月，您通过付钱的方式得到过多少个男性为您提供性服务？________个（没有请填 0） |
| C09你跟男性发生肛交性行为时，你的性角色是：①只做“1”② 只做“0”③两个都有 |
| C10您曾经与女性发生过阴道性交吗?①有②没有 |
| C11在过去的6个月里您与多少位女性发生过性行为？________个（没有请填 0） |
| C12最近6个月跟女性发生性行为时使用安全套的频率如何？①从不②有时（<50％）③经常（>50％）④每次都用 |
| C13最近6个月，你曾与多少个女性发生过一夜情（免费的）？_______________个（没有请填 0） |
| C14您是否在六个月内为了得到钱，为男性或女性提供过商业性性服务(口交或肛交)？ ①是②否 |
| C15.您是否在六个月内为了得到钱，为男性提供过商业性性服务(口交或肛交)？①是②否 |
| C16您为男性提供商业性性服务多长时间了？①>1年，_____年②<1年, _____个月 |
| C17您吸毒吗？（包括海洛因、可卡因、鸦片、大麻、吗啡、冰毒、杜冷丁、K粉(氯氨酮)、摇头丸、麻古）①是②否 |
| C18您注射过毒品吗？ ①是②否 |
| C19您与别人共用过针具吗？ ①是②否 |
| C20最近六个月注射毒品时，你与别人共用针具的频率如何 ①从未共用过②有时共用③每次都共用 |
| C21最近一年，你是否曾被诊断患过性病？①是②否 |
| C22最近一年，你曾被诊断患过何种性病？①淋病②梅毒③生殖道沙眼衣原体感染④尖锐湿疣⑤生殖器疱疹 ⑥其它（请注明） |
| D01您做过艾滋病检测吗？ ①是②否 |
| D02你自己知道检测结果吗？①是②否 |
| E01本次调查是否采血①是②否 |
| E02本次调查未采血原因①既往检测HIV抗体阳性②拒绝采血 |
| E03 HIV抗体检测结果  第一次ELISA初筛①阳性 ②阴性;第二次ELISA复检①阳性 ②阴性; 确认试验结果①阳性②阴性 |
| E04梅毒检测结果 ①阳性②阴性 |
| E05 HCV抗体检测结果，第一次ELISA初筛①阳性②阴性；第二次ELISA复检①阳性 ②阴性 |
