# Supplementary material for: Changes in Prevalence of HIV or Syphilis among Male Sex Workers and Non-Commercial Men Who Have Sex with Men in Shenzhen, China: Results of a Second Survey
Source: PLoS One. 2016 Dec 9;11(12):e0167619. doi: 10.1371/journal.pone.0167619 (PMC5147921; doi:10.1371/journal.pone.0167619)
Supplement: S2 Table — (DOCX) [file pone.0167619.s002.docx]

S2 The survey questions in English

| A01 questionnaire number：＿＿＿­＿ |
| --- |
| A02 date of survey：＿＿＿­＿ |
| A03 sample source：①bar②dancing hall③teahouse④club⑤bathing pool⑥sauna⑦massage  center ⑨other（please specify＿＿＿­＿） |
| Before the survey, we would like to determine whether or not you suit for this survey. In the past six months, have you ever engaged in male sex in Shenzhen or Hong Kong (Including anal or oral sex)?①no（not appropriate respondents）②yes（continue to survey） |
| B01 date of birth：＿­＿day＿＿month＿＿year |
| B02 marital status：①unmarried②married |
| B03 place of domicile： ①Shenzhen ②other（please specify＿＿＿­＿） |
| B04 nationality：＿＿＿­＿ |
| B05 length of stay in Shenzhen: ①<3 months② 3-6 months③7-12 months④1-2years⑤>2years |
| B06 degree of education: ①illiteracy②primary school③junior high school④High school or technical secondary school ⑤college or above |
| B07 current working conditions：①full-time ②part-time job③unemployment④retired ⑤self-employed⑥student⑦student (please specify＿＿) |
| B08 monthly income (RMB)：①no Income ②below 1000③1001－2000④2001－3000⑤3001－4000⑥4001－5000⑦5001－7000⑧7000以上 |
| B09 whether you currently live in shenzhen？①yes②no |
| C01 how do you evaluate your present sexual orientation？①homosexual②heterosexuality③heterosexuality④uncertainty |
| C02 age of your first sex（Including oral sex , vaginal, anal sex with a male or female）？＿＿year |
| C03 gender of the first sexual partner？①male ②female |
| C04 In the past 6 months，how many relationship you have ever been in boyfriend（BF）？______ （no please fill 0） |
| C05 in the past 6 months，How often do you have sex with your boyfriend：average ________ month/times |
| C06 in the past 6 month, the frequency of condom use in the male sex？①se condoms②sometimes（<50％）③sometimes（>50％）④every time |
| C07 in the past 6 months，how many one-night stands male sexual partners did you have？________（no please fill 0） |
| C08 in the past 6 months，How many times have you participated in group sex ( three or more people)？________times（no please fill 0） |
| C09 in the past 6 months， how many men have been provide sexual services for you by way of paying？________（no please fill 0） |
| C10 Anal sex role in male-male sex：①insertive only②receptive only③both |
| C11 have you ever had vaginal intercourse with women?①yes②no |
| C12 in the past 6 month, how many women have sex with you？________（no please fill 0） |
| C13 in the past 6 month, the frequency of condom use in the female sex？①se condoms②sometimes（<50％）③sometimes（>50％）④every time |
| C14 in the past 6 month, how many women had a one-night stand with you（for free）？_______________（no please fill 0） |
| C15 in the past 6 month, Do you have commercial sex services for male or female (oral or anal sex)？ ①yes②no |
| C15 in the past 6 month, Do you have commercial sex services for male (oral or anal sex)？①yes②no |
| C17 how long have you provide commercial sex services for men？①>1 year，_____year②<1year, _____month(s) |
| C18 have you ever taken drugs？（Including heroin, morphine, opium, marijuana, cocaine, methamphetamine, demerol, K powder, ecstasy）①yes②no |
| C19 have you ever taken injected drugs？ ①yes②no |
| C20 have you shared the needles with others？①yes②no |
| C21 while used inject drugs in the past 6 months，how often you sharing needles with others①never share ②sometimes sharing③every time sharing |
| C22 have you ever been diagnosed with a sexually transmitted disease？①yes②no |
| C23 had what disease？ ①gonorrhoea ②syphilis③chlamydial trachomatis④condyloma acuminata⑤herpes progenitalis⑥other（please specify＿＿＿­＿） |
| D01 Had a previous HIV test？①yes②no |
| D02 you know the test results？①yes②no |
| E01 blood collection for this survey?①yes②no |
| E02 the reason for no blood collection for this survey①HIV antibody positive②refuse collection |
| E03 the HIV antibody test results  primary screen with ELISA①positive②negative; second stage certification with ELISA  ①positive②negative; confirmation ①positive ②negative |
| E04 syphilis test results ①positive ②negative |
| E05 HCV antibody detection results  primary screen with ELISA①positive②negative; second stage certification with ELISA①positive ②negative |
